# Woodhall

**Published:** 1888-06-30

**Authors:** 


### AN OLD AND NEW ENGLISH SPA.

BY OUR SPECIAL COMMISSIONER.

BUXTON, Bath, and Harrogate are known to all men, but where and what is Woodhall? The unthinking public, and many who would be deeply offended if they were classed with the unthinking, value a man, or a book, or a place according to the degree of notoriety he or it has attained to in the world. Newton, before his genius had raised him on high, was probably considered a very commonplace mortal by many of his prosperous and untaught neighbours. Perhaps Woodhall may yet prove a Newton among Spas.

It is not an unwise judgment which prompts the medical man to send his patients to distant health-resorts. Pharmacopœial medicines—that is, drugs—are of a strictly limited range of efficacy. They are not to be despised, any more than bread, potatoes, and mustard are to be despised by him who is placed before an uncut, cold sirloin. Those people who affect to contemn all drugs are either entirely destitute of knowledge and experience on the subject, or they may be charitably hoped to be a little "off their heads." Skilfully-used remedies of the drug kind are, in their own place, of a value which cannot easily be exaggerated. But the physician has to deal with many cases in which drugs are of little more avail than brimless hats at midsummer, or dilapidated umbrellas in an April shower. If a man's nervous system be exhausted and worried beyond endurance, it is not physic he wants, but rest and change. If the pressure and strain of family cares have lowered a woman's strength, impaired her appetite, and reduced her to a condition of spiritless debility, she wants new scenes, a new atmosphere, and a life in which domestic worries are unknown. In cases such as these a place like Woodhall, and water like the Woodhall water, may offer the very conditions demanded for immediate relief and gradual restoration to perfect health.

Where, then, and what is Woodhall? It is in Lincolnshire, and is a small village, miles and miles from the "madding crowd" and the "busy haunts of men." It is almost impossible to imagine a place more suggestive of quiet and peaceful repose. The spring, the baths, and the hotel—the latter clean to a degree, and fresh and sweet as early violets —are all embowered in soft and leafy trees, whilst spreading lawns run out in all directions to the fields and woods beyond. The village has long been famous for its mineral spring. Very many years ago a local landed proprietor, digging for other mineral wealth, came, at the depth of 500 feet, upon a decidedly salt spring. In appearance the water is very much like ordinary water, but the miner who first tasted it must have thought it flowed direct from the sea. Woodhall is 18 miles from the sea, and is in the centre of the county. It has certain peculiarities, which may be accounted advantages or disadvantages, according to the taste and requirements of the visitor. The country round about could, by no stretch of imagination, be called hilly. There are certain elevations within a mile or two, but no geographer—not even the local schoolmaster—would dignify them with the name of mountains. Within driving distance are the wolds, which rise to considerable elevations and give variety and interest to the scenery. To some people such a disposition of country is tame and uninteresting; but to others it is the very acme of perfection. It certainly makes walking exercise easy, particularly to those who have weak hearts or who suffer from dyspeptic palpitation. Besides, the level character of the ground may have something to do with the extreme dryness and mildness of the atmosphere. Woodhall is situated on sand and gravel, and has the smallest rainfall of any town or village in England—a fact of very great significance. The air there is almost always bracing, because it is almost always dry; and yet it is not cold. The adjacent rising ground consists of moorland clothed with heather, and pines and fir trees abound as at Bournemouth. So far as atmospheric conditions are concerned, the climate of Woodhall is probably all that can be desired. Its dry, bracing, and mild air will be held to more than compensate for its want of hills—at any rate by a very large number of people in search of health.

The spring, with the baths and hotel, are all situated within grounds of their own of considerable size, and there is a park of seventy acres, the property of the syndicate who hold the baths and hotel, in which visitors may roam or play tennis or cricket, or any other games to their heart's content. Although Woodhall could not at present make any pretence to being excessively "gay," it has no intention whatever of being "dull." It has its own string band of ten or a dozen performers engaged for the whole season, and a very excellent band it is. Standing among the thick shrubs of the hotel garden, or in the woods which extend almost close to the pavilion, the strains of the players, as they discourse soft music, are indescribably soothing and sweet. The violin and the "cello" are the laureates of music, and in forest glades, such as there are around Woodhall, they suggest sylvan dances, and simple, old-world delights, which make bad men wish to be good, and old men grow young again.

The water, however, gives Woodhall its chief right to fame. The Woodhallers will admit that their neighbourhood may be less hilly than some districts of the Alps; they claim, however, that their shops now being constructed by Mr. R. A. Came (architect), in the form of a cloistered bazaar, wlil rival those of Kreuznach or Leamington. "But where, at home or abroad," say they, "will you find mineral water equal to that which flows in perennial streams from the well at Woodhall?" At Kreuznach a very similar water is found, and it is this water which has made the reputation of that town as a fashionable and much-frequented resort. But in the constituents which give the Kreuznach water its medicinal reputation, the Woodhall water is more than double the strength of its Continental competitor. There is nearly twice the quantity of bromine and more than six times the quantity of iodine in the Woodhall water that are found in any other European spring. In order to gain experience from the system so successfully carried out at Kreuznach, the syndicate have directed a thorough examination of this by an expert, and have determined that the Kreuznach system shall be adopted at Woodhall, thus introducing it into England for the first time.

For the convenience of medical readers we subjoin an analysis of the Woodhall water. Four different analyses have been made—by Dr. Franklyn, F.R.S., Mr. West (of Leeds), Dr. Ziurck, and Professor Wanklyn, M.R.C.S., all of which are in substantial agreement as to the constituents of the water. One gallon contains: Chloride of sodium, 1330·00 grains; chloride of calcium, 111·00; chloride of magnesium, 91·20; carbonate of soda, 10·00; sulphate of soda, ·30; nitrate of soda, ·55; free iodine, ·20; iodine (as iodates), ·20; iodine (as iodides), ·40; bromine (as bromides), 3·40; peroxide of iron, traces. For a bromo-iodine water Woodhall may be considered the finest spa in Europe. This kind of water is eminently suited for glandular affections. It has been recommended by the medical profession in all forms of gout and neuralgia, eczema and allied diseases of the skin, disorders of the liver and kidneys, impaired digestion and its consequences. The *British Medical Journal* pronounces Woodhall "One of the most valuable iodine spas in Europe." The *Lancet* echoes this statement, and declares it "One of the most valuable and remarkable spas in Europe." Dr. Wanklyn says, "It will prove one of the best mineral waters in the world." Dr. Granville affirms that it is "a most remarkable mineral water, endowed with the most important chemical properties, which, in the hands of a skilful and judicious practitioner, may be made instrumental in curing some of the diseases mostly prevalent in England, including disordered digestion, symptomatic rheumatism, rheumatic gout, and gout itself."

Here, then, we have, within easy reach of London and many other large English towns, a mineral water of unique value, in a village of rare simplicity and repose, associated with a mild but bracing and equable climate, with every modern convenience for carrying out completely the famous Kreuznach system; with comforts and conveniences such as only an English hotel and English homes can furnish; with park, tennis lawns, a good band, easy walks, and a variety of advantages which the chronic invalid or the seeker of rest and change so much appreciate. The syndicate who, with much courage and public spirit have made these resources available, deserve all the encouragement and support they have so courageously determined to obtain. The Great Northern Railway Company is now building a new and enlarged station, and reduced fares are promised for the return London journey. For our part, we hope to see Woodhall Spa, within the next decade, take rank among the first of English watering-places.

